# Surgical outcomes in patients with optic disc pit maculopathy: does peeling the ILM lead to better outcomes?

**DOI:** 10.1007/s10792-020-01524-z

**Published:** 2020-08-01

**Authors:** Helena Wagner, Amelie Pielen, Hansjürgen Agostini, Daniel Böhringer, Wolf Alexander Lagrèze, Julia Biermann

**Affiliations:** 1 Eye Center at Medical Center, University of Freiburg, Killianstrasse 5, Freiburg, Germany; 2 Faculty of Medicine, University of Freiburg, Freiburg, Germany; 3 Hannover Medical School, University Eye Hospital, Carl-Neuberg-Str. 1, Hannover, Germany; 4 Department of Ophthalmology, University of Muenster Medical Center, Domagkstrasse 15, 48149 Muenster, Germany

**Keywords:** Optic disc pit, Maculopathy, ILM-peeling, Pars plana vitrectomy, Gas tamponade

## Abstract

**Purpose:**

Optic disc pits (ODPs) are rare congenital anomalies. Several patients develop optic disc pit maculopathy (ODP-M): visual impairment caused by intra- and/or subretinal fluid. Treatment mode remains controversial. This study was designed to investigate the effectiveness of pars plana vitrectomy (PPV) and gas tamponade with or without internal limiting membrane (ILM)-peeling in improving visual acuity and reducing subretinal fluid in ODP-M patients.

**Methods:**

We retrospectively reviewed the charts of 16 patients who underwent surgery for ODP-M from 2002–2015. Six patients underwent PPV with gas tamponade (group 1); ten patients additionally received ILM-peeling (group 2). Pre- and postoperative visual acuity and central retinal thickness (CRT) were compared between groups, as well as retinal morphology and the number of secondary vitrectomies and complications.

**Results:**

Median visual acuity improved by 2 ETDRS lines in both groups (*p* = 0.713, Mann–Whitney U test). Median CRT decreased by 426.5 µm and 460 µm (*p* = 0.931). One patient in group 1 underwent repeat vitrectomy for persistent retinoschisis. Three patients in group 2 required repeat vitrectomy: two to treat a macular hole, one for peripheral retinal holes with retinal detachment.

**Conclusion:**

In our cohort, PPV with gas tamponade proved to be an effective first-line treatment for ODP-M. Additional ILM-peeling did not give a significant benefit in this study.

## Introduction

Optic disc pits (ODPs) were first described by Wiethe in 1882 as 2 black excavations within the optic disc of a 62-year-old woman [1]. Today, ODP is known to be a rare congenital anomaly of the optic nerve head (ONH) as 1 in 11 000 exhibits ODP [2]. Funduscopically, ODP appears as grey, yellow or black oval excavations of the ONH that occur temporally in most cases [3]. Usually they are limited to one eye; 15% occur bilaterally [4].

Several histopathological reports describe ODP as a herniation of the dysplastic retina into a pocket lined with collagen. The herniation extends up to the subarachnoidal space through a defect in the lamina cribrosa [4, 5]. Although ODP can occur sporadically, autosomal-dominant heredity is also described [6].

Clinically, ODP usually remains asymptomatic and patients exhibit a normal visual acuity (VA). However, ODP can be associated with variable visual field defects, the most prevalent of which are paracentral arcuate scotomas connected to an enlarged blind spot [7]. Furthermore, a significant number of eyes with ODP develop serous macular detachment associated with intra- and/or subretinal fluid and changes in the pigment epithelium. These changes lead to a significant impairment of VA [7, 8]. In particular, the occurrence of subretinal fluid seems to be associated with persistent visual impairment [9]. This complication is known as optic disc pit maculopathy (ODP-M).

ODP-M commonly occurs in the third or fourth decade of life, but has been described in children as well [10, 11]. Two conflicting theories exist regarding the aetiology of ODP-M. One theory suggests an association with posterior vitreous detachment. Although the ODP existed congenitally, ODP-M occurs at an older age [12]. Different findings support the theory of a traction-related cause of maculopathy. Studies with optical coherence tomography (OCT) have found vitreous strands over the ODP and the rupture of a membrane over the ODP, possibly due to traction. This finding has been presumed to play a role in the development of ODP-M [13]. The other theory suggests that ODP-M is associated with a reversal between intracranial and intraocular pressure [14], and possibly prepapillary liquefaction [15].

The source of the subretinal fluid remains controversial as well. According to some studies, the fluid could emerge from the optic nerve subarachnoid space [5]. Others suspect leaky vessels at the base of the ODP as a possible source [16]. A vitreous origin of the fluid is more likely, as ink injected intravitreously into the eyes of collie dogs with ODP migrated into their subretinal space. Nonetheless, a direct connection could not be demonstrated by OCT in that study. Also, no important components of the vitreous (glycosaminoglycans) could be found in the subretinal fluid [7]. However, electron microscopy on a cadaveric human eye with ODP-M found holes over the ODP connecting the intraretinal space with the vitreous [17]. Another histopathologic study on two human eyes with ODP-M detected mucopolysaccharides, another component of the vitreous, inside the ODP [18]. Furthermore, a number of studies observed the passage of gas or silicone oil from the vitreous cavity to the subretinal space in eyes with ODP [19, 20], and in some cases, the subretinal fluid was successfully drained through the membranous defects overlying the pit [21].

Another probable source of the fluid is the subarachnoidal space. A study from 2006 reported the intracranial migration of silicon oil injected during a vitrectomy in an eye with ODP [22]. A recent study found the composition of electrolytes and proteins in drained subretinal fluid to be similar to that in cerebrospinal fluid [23].

ODP-M can lead to a severe decrease in VA, which reduces quality of life due to the loss of reading ability [24]. Careful consideration of the different treatment options is crucial to prevent, reduce or cure visual impairment related to ODP-M. Despite the new findings concerning its pathogenesis, no consensus has yet been reached regarding the best treatment for ODP-M. Historically, a conservative approach including bed rest and the covering of both eyes was followed with mixed results, but today surgical interventions are favoured. The techniques employed are manifold, among them macular buckling [25], optic nerve sheath fenestration, the drainage of the subretinal fluid [26] and, far less commonly, the use of autologous platelet concentrate [27], autologous fibrin [28] or inverted autologous internal limiting membrane (ILM) to cover the ODP, which seems to be a promising technique [29–31]. More common techniques are peri- or juxtapapillary laser coagulation (in the hope of forming a barrier between the ODP and the peripapillary retina) and gas tamponade. While the former was not very successful as an exclusive therapy [32], the gas tamponade was at least able to improve VA in 4 of 8 patients [33]. The combination of these techniques with pars plana vitrectomy (PPV) appears promising. In some cases, peeling of the ILM is additionally performed.

The goal of this study was to assess the surgical outcome of PPV and gas tamponade with or without ILM-peeling. The improvement of VA and reduction in subretinal fluid were analysed for both surgical techniques.

## Materials and methods

### Ethics statement and study design

**Fig. 1:**
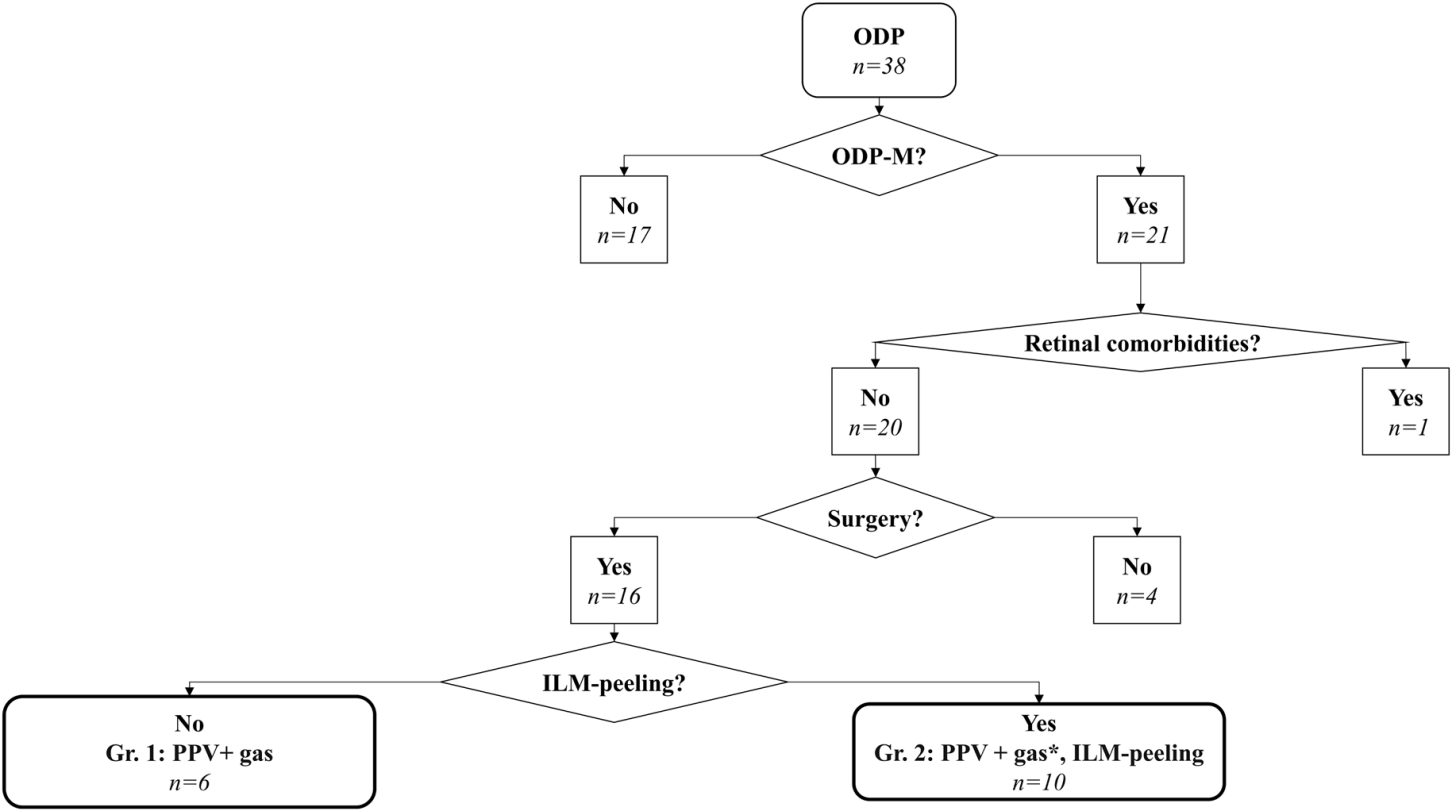
Distribution of patients. Flow chart showing the inclusion process of our patients; *no gas for *n* = 1. *ODP-M*, optic disc pit maculopathy, *PPV* pars plana vitrectomy; n, number of patients who were operated on following the respective technique. Microsoft PowerPoint was used to create this figure

The monocentric retrospective study was conducted at the Eye Centre Freiburg with approval from the Ethics Committee of the University of Freiburg (permit number 64/13) and adhered to the tenets of the Declaration of Helsinki. All data were fully anonymized before they were accessed. Therefore, and because this is a retrospective, intradepartmental, non-interventional study, the local ethics committee waived the requirement for informed consent. Digital hospital archives at the Eye Centre of the University Hospital Freiburg were reviewed for patients with ODP between 2002 and 2015. Figure 1 illustrates our selection process. The total number of patients diagnosed with ODP was 38. Among these patients, 21 developed fluid in/under their retina due to ODP-M and experienced the related reduction in VA. One patient was excluded due to retinal co-morbidities. Sixteen out of 20 patients were surgically treated with PPV, 15 with additional gas tamponade and 10 with additional ILM-peeling (see Fig. 1). To investigate the effectiveness of PPV and gas tamponade with or without ILM-peeling, two groups were established. Group 1 included the six patients surgically treated with PPV and gas tamponade only. Group 2 included the ten patients treated with PPV, gas tamponade and ILM-peeling. Four patients were not operated on (*n* = 2 did not consent to the operation, *n* = 1 because of uncertain prognosis, and *n* = 1 decided against surgery because of a lack of progression of the ODP-M). The outcomes of the eyes with ODP-M that underwent surgery were further analysed in the presented study.

### Data collection and surgery

Data were extracted from medical reports, and OCT images were analysed retrospectively. The collected data included preoperative and postoperative VA, presence or absence of intraretinal fluid, serous detachment or macular holes, duration of preoperative symptoms, age at surgery, preoperative and postoperative central retinal thickness (CRT), surgical technique, number of surgical interventions required and complications. The postoperative best-corrected VA was defined as that recorded at the last follow-up after the last performed surgery. The surgical techniques included 20- or 23-gauge PPV with gas tamponade using either sulphur hexafluoride (SF6) in 13 cases or air in 2 cases and peeling of the ILM in 10 cases (Table 1).

**Table 1:**
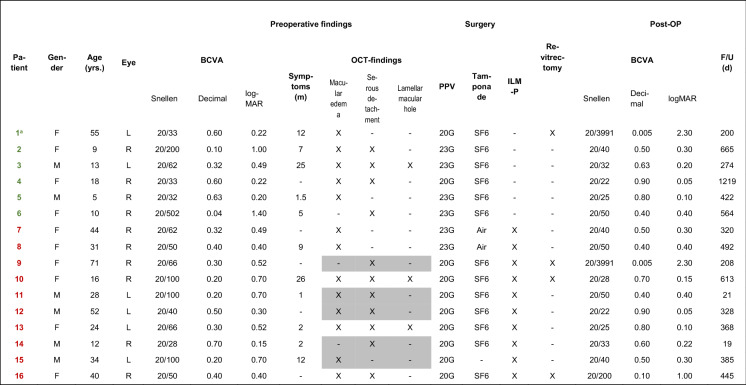
Clinical characteristics and surgeries performed on ODP maculopathy patients

*F* female, *M* male. Age at the time of surgery (years). *R* right eye, *L* left eye. Best-corrected visual acuity (BCVA). Duration of symptoms before surgery in months. *OCT*-findings, optical coherence tomography; PPV, gauge size used for pars plana vitrectomy; SF6, gas tamponade with sulphur hexafluoride; *ILM-P* internal limiting membrane peeling; F/U = Follow-up in days

^**a**^Patient 1 with additional peripapillary laser coagulation. The OCT findings marked in gray have been retrieved from the clinical report, but their original OCT images could not be retrieved and reanalysed

For 14 patients, a complete posterior vitreous detachment (PVD) could be achieved during PPV. For 1 patient (patient No. 16 in Table 1), only a partial PVD was possible, and for patient No. 9, PVD was already present at the beginning of the vitrectomy.

Patient 1 in Table 1 was additionally subjected to peripapillary laser coagulation. This patient exclusively received drainage of subretinal fluid through a pre-existent retinal hole next to the temporal ODP.

The surgeries were performed by HA, WL and another experienced surgeon.

For 11 patients, images were available with Spectralis OCT (Heidelberg Engineering, Heidelberg, Germany). The OCT images of the remaining 5 patients could not be retrieved, as they were operated on before we began digital storage of imaging in our archives. However, the overall findings were documented in their medical reports. CRT was determined by automatic segmentation and was manually corrected if needed to ensure accuracy.

VA was converted to logMAR for statistical analysis. Clinical acuity categories “hand motion” and “counting fingers” were converted to logMAR as described in an earlier study [34].

### Statistics

Fisher’s exact test was used to analyse categorical variables. As the data were not normally distributed in the sample group and the groups were independent, variables were analysed using the Mann–Whitney U test. To describe general tendencies, the median and quartiles were calculated to accommodate the small sample size. P-values below 0.05 were considered statistically significant.

## Results

### Clinical Characteristics

Baseline data and the respective surgeries performed on the 16 patients are shown in Table 1. Group 1 consisted of 4 women and 2 men, while group 2 comprised 6 women and 4 men (*p* = 1.000, Fisher’s exact test). In group 1, the median age was 11.5 years (range: 5–55 years), while in group 2 it was 32.5 years (range: 12–71 years), a difference not reaching statistical significance (*p* = 0.056, Mann–Whitney U test). In 15 cases, the ODP was located temporally; one of them occurred superotemporally and 4 occurred inferotemporally. In one case of ODP-M, the exact localization was not documented. There were no statistically significant differences between the groups regarding the location of the ODP.

All the patients had a history of rapid progressive visual impairment following ODP-M. The median preoperative VA of all our patients was 20/62 (0.49 logMAR; low quartile: 0.24 logMAR; high quartile: 0.70 logMAR). In group 1, the median preoperative VA was 20/46 (0.36 logMAR); in group 2, it was 20/65 (0.51 logMAR). The difference between the groups was not statistically significant (*p* = 0.792, Mann–Whitney U test). The interval of time between the appearance of the first symptoms of ODP-M and the first surgery could be determined for 11 patients and had a median duration of 7 months (low quartile: 2 months; high quartile: 12 months). The duration was median 12 months in group 1 and 5.5 months in group 2 (*p* = 0.792).

### Preoperative retinal morphology

**Fig. 2:**
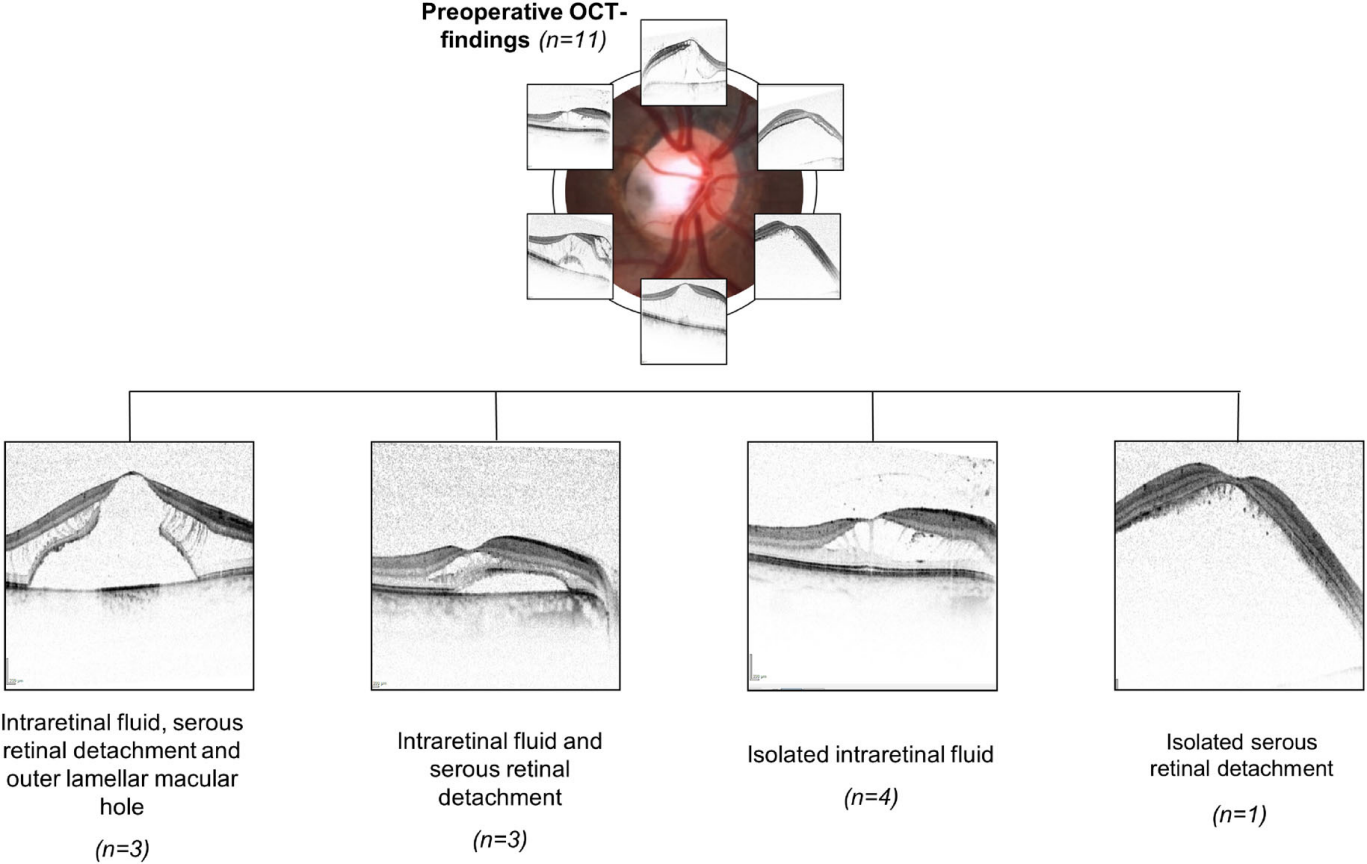
Preoperative retinal changes. Flow chart with exemplary OCT images of some of our patients illustrating the preoperative OCT findings. Microsoft PowerPoint was used to create this figure

Figure 2 summarizes the preoperative OCT findings. Intraretinal fluid in the outer retina, especially in the outer nuclear layer, was found in 10 out of 11 patients. Among these 10 patients, 6 had additional fluid in the ganglion cell layer or retinal nerve fibre layer.

Seven of the 11 patients showed a connection between the fluid reservoir and the ODP. These 7 patients include 3 cases where preoperative OCT examinations did not include images of the ODP, thus requiring an additional viewing of the postoperative OCT images to evaluate the existence of a connection. The median preoperative CRT was 809 µm (lower quartile: 745 µm; upper quartile: 948 µm, *n* = 11). In group 1, the median preoperative CRT was 796 µm; in group 2, it was 809 µm (*p* = 0.537).

### Comparison of surgical techniques

Before 2012, 20G vitrectomies were performed. From 2012 onwards, only 23G vitrectomies were carried out (Table 1).

To facilitate the ILM-peeling, the ILM was dyed prior to peeling. Until 2009, indocyanine green was used, from 2009 on exclusively brilliant blue. Indocyanine green was used in six, brilliant blue in four cases.

### Comparison of postoperative results of vitrectomy and gas tamponade with or without ILM-peeling

The postoperative results were analysed for both surgical groups to evaluate the benefit of additional ILM-peeling in reducing intra- and subretinal fluid and improving VA in ODP-M patients.

**Fig. 3:**
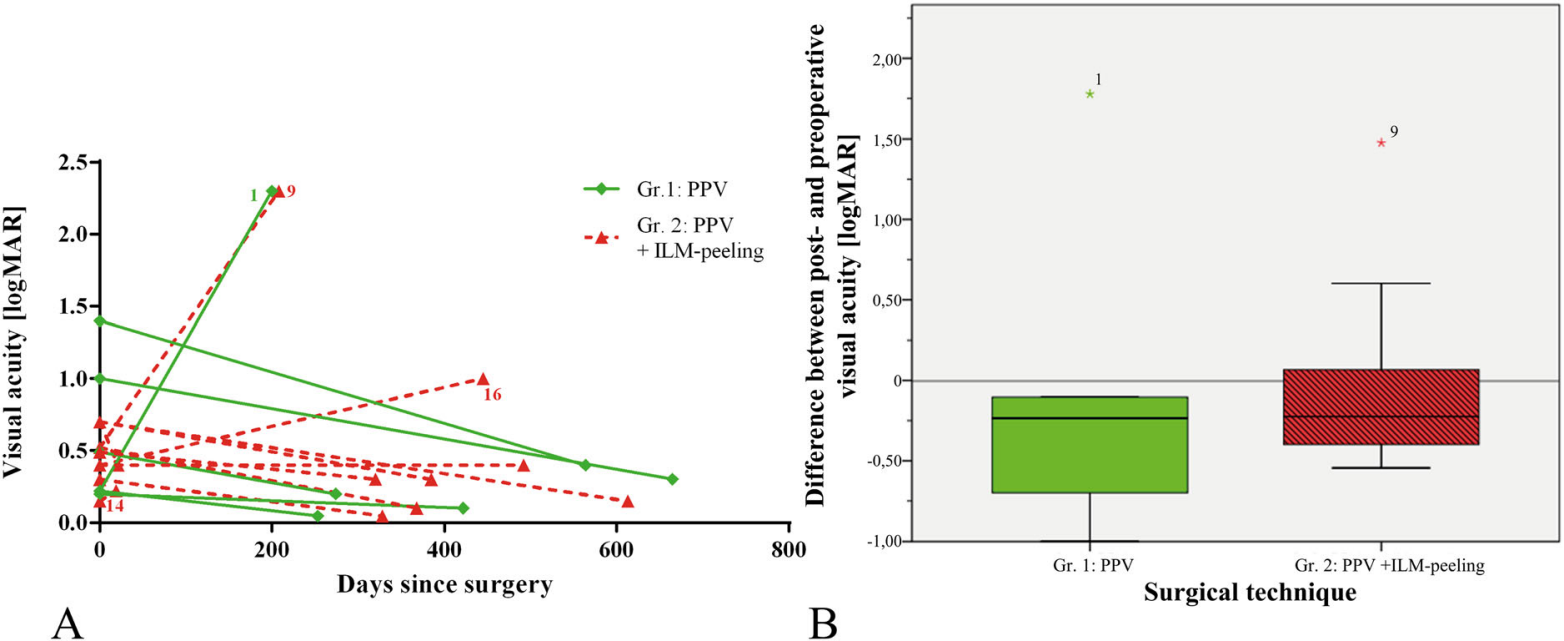
Postoperative visual acuity change. **a** This graph only represents the respective logarithmic visual acuity of the patients before surgery (days since surgery = 0) and the visual acuity at their last visit represented in days after surgery for the sake of clarity. A downward pointing line shows an improvement, as the visual acuity is represented in logMAR. The reasons behind the unfortunate development of the visual acuity of patients 1, 9 and 16 are given in Table 2. Patient 14 had only a very short follow-up of 19 days. **b** This boxplot shows the median difference between post- and preoperative visual acuity as the thick line; the boxes show low and high quartiles for group 1 and group 2 (*p* = 0.713). The star marks single outliers. The median logarithmic visual acuity reduction was 0.24 logMAR in group 1 and 0.22 logMAR in group 2. Thus, both groups experienced a median visual improvement of over 2 lines on the ETDRS scale. The GraphPad Prism software version 6.01 was used to create Fig. 3a, and the version 23.0 of the IBM SPSS statistics software was used to create Fig. 3b

As shown in Fig. 3a, 68.75% (11 out of 16) of all patients showed an improvement of VA at their last postoperative follow-up visit. In group 1, 5 out of 6 patients experienced improvement of their VA, while in group 2, 6 out of 10 patients experienced improvement (*P* = 0.753).

Patient No. 8 (see Table 1) did not experience an improved VA, although macular fluid decreased postoperatively.

As shown in Fig. 3b, the median logarithmic VA improvement was 0.24 logMAR in group 1 and 0.22 logMAR in group 2. Thus, both groups experienced a median visual improvement of more than 2 lines on the ETDRS scale. The Mann–Whitney U test showed no significant difference between the 2 groups (*p* = 0.713). When comparing the postoperative VA of the 2 groups, group 1 demonstrated a median VA of 20/36 (0.25 logMAR; lower quartile: 0.08 logMAR; upper quartile: 0.87 logMAR), group 2 demonstrated a median VA of 20/40 (0.30 logMAR; lower quartile: 0.14 logMAR, upper quartile: 0.55 logMAR), and there was no significant difference between the two (*p* = 0.713).

**Fig. 4:**
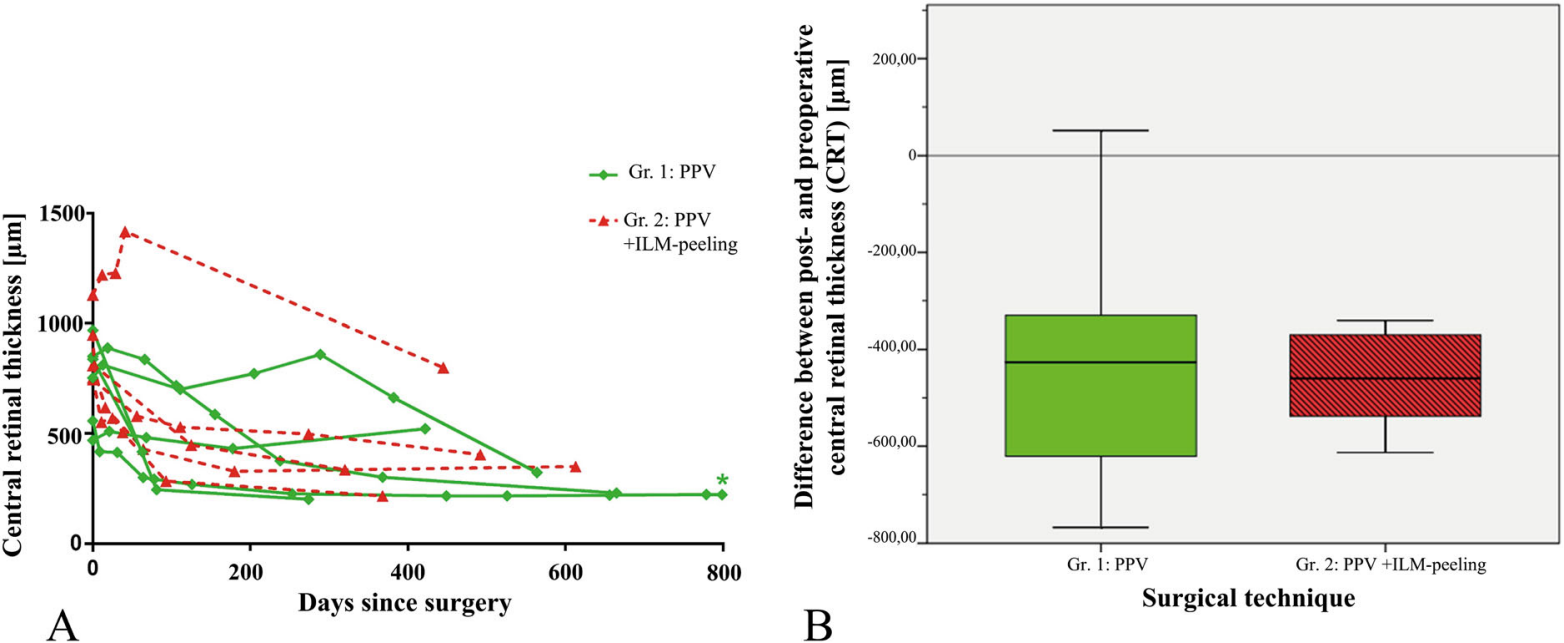
Postoperative CRT change **a** This graph represents the time course of CRT after surgery (*n* = 11); *Patient 4 (see Table 1) last follow-up day 1219 with CRT 227 µm. This graph represents the respective central retinal thickness [CRT] of the patients before surgery (days since surgery = 0) and the CRT at their follow-ups represented in days after surgery. **b** This boxplot shows the median difference between post- and preoperative central retinal thickness as the thick line; the boxes show low and high quartiles for group 1 and group 2 (*p* = 0.931). The median CRT reduction in group 1 was 427 µm (low quartile: 235 µm; high quartile: 658 µm), and the median CRT reduction in group 2 was 460 µm (low quartile 356 µm; high quartile: 576 µm). The GraphPad Prism software version 6.01 was used to create Fig. 4a, and the version 23.0 of the IBM SPSS statistics software was used to create Fig. 4b

As shown in Fig. 4a, 90.9% (10 out of 11) of the patients whose CRT could be measured (*n* = 11) showed a postoperative reduction in CRT of at least 330 µm during follow-up. In group 1, 5 out of 6 patients experienced a reduction, and in group 2, 5 out of 5 patients had reduced CRT (*p* = 0.093). CRT postoperatively decreased in all of the patients of group 2 and in all but one patient in group 1. This patient also experienced persistent maculopathy with intraretinal fluid relative to the vitrectomy.

Overall CRT was noted as having been reduced postoperatively by 431 µm (lower quartile: 341 µm; higher quartile: 613 µm). As shown in Fig. 4b, the median CRT reduction in group 1 was 427 µm (lower quartile: 235 µm; upper quartile: 658 µm), and the median CRT reduction in group 2 was 460 µm (lower quartile 356 µm; upper quartile: 576 µm; *p* = 0.931).

The median last postoperative CRT in group 1 was 276 µm (lower quartile: 221 µm, upper quartile: 442 µm) and 349 µm (lower quartile: 276 µm, upper quartile: 602 µm) in group 2 (*p* = 0.537).

Complete fluid resolution was only achieved in 31% of patients (5 out of 15: patients 2, 4, 10, 12, 13) with no significant difference between the groups (group 1: 2 out of 6; group 2: 3 out of 10; *p* = 1.000). The overall median time to resolution was 526 days (range: 328–665 days) (group 1: 596 days; group 2: 368 days, *p* = 0.400).

### Postoperative retinal fluid resorption

The subretinal fluid disappeared before the intraretinal fluid disappeared in 4 patients. Preoperative intraretinal fluid, serous retinal detachment and a lamellar macular hole were observed in 3 out of these 4 patients. The fourth patient (No. 2 as determined in Table 1) had initial intraretinal fluid and serous detachment, and developed a lamellar macular hole postoperatively.

**Fig. 5:**
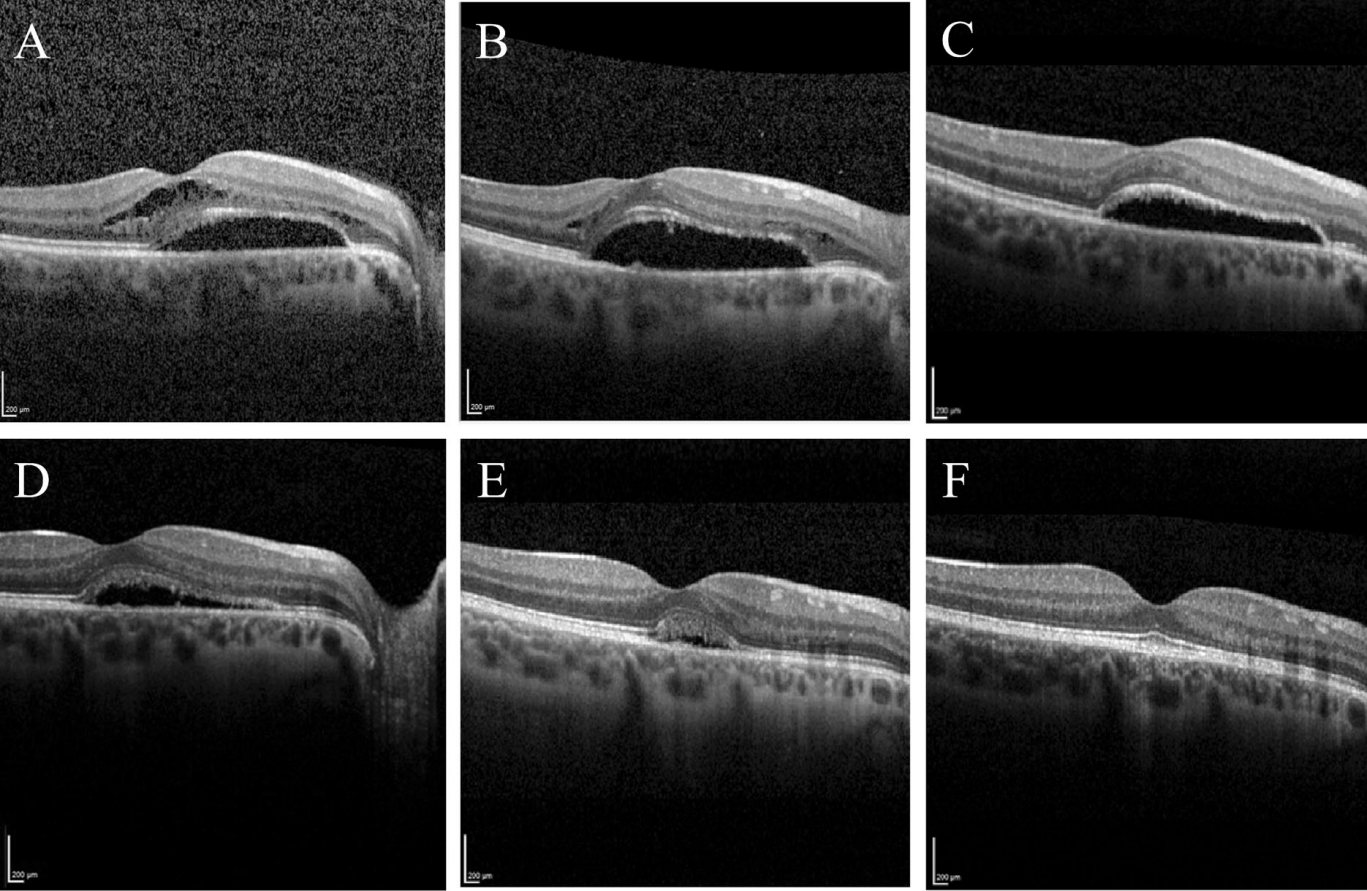
Postoperative OCT findings of the right eye of patient 4. Postoperative OCT findings of the right eye of an 18-year-old female patient who was subjected to 20-gauge vitrectomy and gas tamponade with SF6 for optic disc pit maculopathy. **a** Preoperative OCT image: retinal detachment with subretinal fluid and intraretinal fluid in the outer nuclear layer. Preoperative visual acuity: 20/36. **b** Day 9 after surgery: reduction in the intraretinal fluid. **c** Day 78: complete absorption of the intraretinal fluid. **d** Day 126: reduction in the subretinal fluid. **e** Day 253: further reduction in the subretinal fluid. **f** Day 526: complete resolution of the subretinal fluid and reattachment. Last postoperative visual acuity: 20/27. Microsoft PowerPoint was used to create this figure

The patients with intra- and subretinal fluid without pre- or postoperative lamellar macular hole formation showed a different fluid resolution sequence. Patient 4 first demonstrated a complete resorption of intraretinal fluid before the subretinal fluid was absorbed (see Fig. 5) and patient 16 showed a reduction in subretinal fluid, but an increase in intraretinal oedema (for patient numbers, refer to Table 1). Thus, the sequence of fluid resolution differed between groups and was independent from the surgical technique used.

### Repeat vitrectomies and adverse events

Table 2 shows the complications from the different surgical techniques used to treat ODP-M and details the 4 repeat vitrectomies that had to be performed. The complications included postoperative cataract, temporarily raised intraocular pressure, and lamellar and full-thickness macular holes.

**Table 2:** Complications and re-vitrectomies after surgeries

	Vitrectomy, gas tamponade and without ILM-peeling (*n* = 6)	Vitrectomy and ILM-peeling (*n* = 10)	Total (*n* = 16)
Raised intraocular pressure	1	1	2
Cataract*	1	1	2
Development of lamellar macular hole	1	2	3
Re-vitrectomy for			
New postoperative full-thickness macular hole	0	2^b, c^	2
Persistent retinoschisis	1^a^	0	1
Persistent retinal detachment with peripheral retinal holes	0	1^d^	1

This table shows the absolute number of patients having the respective complication.

*Patients who developed visually significant cataract after initial pars plana vitrectomy, necessitating phacoemulsification surgery that was performed prior to the last follow-up visit. The surgeries, which had to be performed additionally, included.

^a^Vitrectomy with C3F8 gas tamponade, ILM-peeling, phacoemulsification and posterior chamber lens implantation to treat persistent retinoschisis and cataract of patient 1

^b^Vitrectomy, epiretinal peeling, the injection of autologous whole blood, gas tamponade, phacoemulsification with posterior chamber lens implantation for macular hole and cataract of patient 9

^c^Vitrectomy, gas tamponade and autologous whole blood for macular hole of patient 10 ^d^First cerclage and cryopexy, followed by a vitrectomy and gas tamponade for persistent retinal detachment due to peripheral retinal holes of patient 16. The numbers attributed to the patients correspond to the numbers in Table 1

Each group included one patient who developed transient rises of intraocular pressure and one patient who developed a visually significant complicating cataract necessitating phacoemulsification surgery. Furthermore, a lamellar macular hole occurred in 1 patient in the first group and 2 patients in the second group.

Overall, 4 patients needed repeat vitrectomy. In the group without ILM-peeling, only one patient had to be subjected to a second vitrectomy due to persisting retinoschisis, while in the group with ILM-peeling, 3 patients needed repeat vitrectomy. Two of the patients had developed a new, postoperative, full-thickness macular hole, and the other one suffered from persistent retinal detachment due to peripheral retinal holes. Details about the second surgeries can be found in the legend of Table 2.

### Surgical techniques

The visual and anatomical outcome did not vary significantly between the different gauge sizes (20G or 23G) used during PPV (postoperative visual acuity *p* = 0.958; difference between postoperative and preoperative visual acuity *p* = 0.368; difference between post- and preoperative central retinal thickness *p* = 0.537, respectively).

There was not a significant difference between the outcomes of the different dyes (indocyanine green or brilliant blue) used to peel the ILM (postoperative visual acuity *p* = 0.476; difference between postoperative and preoperative visual acuity *p* = 0.257; difference between post- and preoperative central retinal thickness *p* = 1.000, respectively).

### Patients with ODP-M without surgical intervention

As Fig. 1 shows, four patients diagnosed with ODP-M at our institution were not surgically treated. Their median age at first presentation was 23.5 years (range: 19–73 years) and their median VA was 20/63 (0.50 logMAR, range: 0.22–0.70 logMAR). Three of them were re-examined later. Follow-up on these three ranged from 56 days to 18 years. One patient showed an increase in subretinal fluid and a stable VA (20/36) after 56 days. Another patient also showed an increase in subretinal fluid and a VA which had decreased by 6 lines on the EDTRS scale after 1195 days. Her VA at her last follow-up was 20/200. The patient with a follow-up time of 18 years experienced complete fluid resolution within 10 years after the first visit, but the VA had similarly decreased by 6 lines on the EDTRS scale and was reduced to 20/400. The reattached retina was atrophic in the area all around the ODP and showed multiple scars in the retinal pigment epithelium.

## Discussion

The contents of this retrospective, monocentric study can be summarized as follows:

A) 16 patients underwent PPV due to ODP-M during the period of observation (14 years), with 6 patients undergoing combined gas tamponade and 10 patients undergoing combined gas tamponade and ILM-peeling. B) The patients of both treatment groups experienced a comparable increase in VA and decrease in CRT, showing no statistically significant benefit of additional ILM-peeling. C) 11 patients (~ 69%) observed a significant and persistent recovery of ODP-M after a single surgery, 4 patients could not be successfully treated and required re-vitrectomy, and 1 patient remained stable. D) The occurrence of full-thickness macular holes postoperatively was a complication exclusive to the peeling group.

As ODP-M is a rare disease, treatment strategies are inconsistent all over the world, and the implementation of comparable studies is challenging. Therefore, case series have been used to gain experience with this disorder. PPV proved to be useful in treating ODP-M at the international level. However, it was modified inhomogeneously by, e.g., gas tamponade, laser coagulation, fluid drainage or ILM-peeling. The reduction in vitreous traction might not be the only explanation for the visual improvement after vitrectomy and gas tamponade. In addition, the shift of subretinal fluid either back into the subarachnoidal space [4, 18, 35] or into the replaced vitreous space [17] after gas tamponade may be involved. In our institution, PPV with gas tamponade is the standard for the treatment of ODP-M, which is combined with ILM-peeling at the discretion of the surgeon. In the presented cohort, we analysed the importance of additional ILM-peeling and saw no clinically significant difference regarding functional or anatomical outcome.

A number of studies demonstrated good anatomical and visual outcomes after vitrectomy combined with ILM-peeling and other techniques [36–38]. Only a few other studies explicitly evaluate ILM-peeling. One study, comparing 5 patients treated with PPV with 4 patients additionally treated with ILM-peeling, found the postoperative central macular thickness to be significantly lower in the peeling group; however, the postoperative VA did not differ significantly between the groups [39]. None of the other studies confirmed this finding or demonstrated any other significant benefit of ILM-peeling [40–42].

In this cohort, full-thickness macular holes developed only in patients of the ILM-peeling group. The occurrence of full-thickness macular holes after additional ILM-peeling to treat ODP-M was reported similarly by other groups [38, 43, 44]. To prevent the formation of macular holes, a new technique was described in a case report in 2017, combining vitrectomy, fovea sparing internal limiting membrane flap and C3F8 tamponade [45]. The patient was treated successfully and did not develop a full-thickness macular hole.

However, in a recent study, two out of 11 patients developed paramacular holes during the process of posterior vitreous detachment without ILM-peeling. This finding might suggest that even without ILM-peeling the tractional forces during vitreous detachment might favour retinal hole formation. According to the authors, these tractional forces might be reduced by preoperative intravitreal Ocriloplasmin injection [46]. Other risk factors for deterioration of visual acuity were the need for additional vitrectomies due to persisting intraretinal fluid. Steel et al. reported 3 similar cases that initially failed to improve after the first vitrectomy combined with gas tamponade and in some cases peripapillary laser or ILM-peeling. They showed persistent lack of visual success even after a second surgery [42]. Reoccurrences of ODP-M after initial success of the first vitrectomy with gas tamponade have been reported in other studies, but reappearances remain few and the retina has usually been reattached after a second intervention [42, 47].

Our study showed that vitrectomy in combination with gas tamponade with and without ILM-peeling leads to a visual improvement in 68.75% of all cases. Overall, the patients showed a relevant improvement of VA with a median visual improvement of over 2 lines on the ETDRS scale and an overall median postoperative VA of 20/40 (0.301 logMAR) [48]. A study following 11 ODP-M patients treated with vitrectomy, posterior vitreous detachment and gas tamponade showed an even bigger improvement of nearly median 4 lines on the EDTRS scale postoperatively [49], as did a study including 9 patients with vitrectomy and gas tamponade with or without ILM-peeling [39]. Other studies evaluating the benefit of vitrectomy combined with different techniques demonstrated a similar [43, 44] or even higher postoperative improvement in VA [40, 47] than our study. Thus, vitrectomy in combination with gas tamponade seems to lead to a relevant visual improvement.

Furthermore, of all the patients whose CRT could be measured (*n* = 11), 90.9% showed a postoperative reduction in CRT. The overall CRT decreased by median 431 µm, indicating a reduction in intra- and subretinal fluid with a final median CRT of 335 µm. In another study, a group composed of 21 patients who underwent vitrectomy combined with gas tamponade showed a median postoperative central macular thickness of 277 μm [41], while the study including the 9 patients with vitrectomy and gas tamponade with or without ILM-peeling shows a median postoperative central retinal thickness of 235 µm [39]. In summary, vitrectomy in combination with gas tamponade and/or ILM-peeling is successful in reducing CRT and thus, in our case, intra- and subretinal fluid.

Complete resolution of retinal fluid could only be confirmed in 5 patients (31.25%). However, the low number of eyes with complete resolution of retinal fluid could be due to an insufficient follow-up time. A quarter of the patients were followed for under 225 days, but in our study the median resolution time for the intraretinal fluid after vitrectomy was found to be 526 days. The other studies with similar techniques showed a higher reattachment rate of 71.4% [41] or even 90.9% [49], with a time to resolution of mean 395 ± 346.8 days or 304 ± 118.6 days (median 365 days), respectively. Another study evaluating the benefit of vitrectomy combined with different techniques for 32 ODP-M patients demonstrated an equally long time to resolution with median time to attachment of 416 days and a mean reattachment time of 768 ± 163 (SE) days [40]. One should therefore expect a long recovery time after surgery.

### Limitations

The study limitations include its retrospective design. Spontaneous improvement cannot be ruled out. Furthermore, there were only six study patients in the group without ILM-peeling. Insufficient statistical power due to small sample sizes potentially led to difficulties in detecting significant differences between the two groups. Another weakness might be an insufficient follow-up time for some of our patients. As our study found the median fluid resolution time after vitrectomy to be 526 days, follow-up times under a year might be too short to show the final effects and results of our surgical treatment for certain patients. In addition, numerous confounding factors must be considered. Between the patients there were clear differences between age, size of the access and different dyes used in ILM-peeling. However, the difference between the characteristics was not significant in our investigations. Different surgeons and different forms of optic disc pit maculopathy can be additional influencing factors. Unfortunately, due to the rarity of optic disc-related maculopathy, a study with more uniform surgical techniques and patients outside a multicentred and prospective setting is not possible. Due to the low incidence of ODP-M, retrospective data over 13 years were evaluated to obtain the patient numbers presented here. A variation of surgical techniques over this period (23G PPV from 2012 on and brilliant blue from 2009 on) cannot be avoided due to new developments in medical technology.

While a number of studies analysing the surgical outcome of PPV with or without ILM-peeling have been conducted in the past, the number of patients was small (not more than 13 [38, 43, 44, 47, 50] and only one study compared patients treated with and without ILM-peeling [39]. This study from 2015 compared patients treated with either PPV (*n* = 5) or PPV with ILM-peeling (*n* = 4) gathered over the course of nearly 14 years. Three retrospective multicentred studies analysing the surgical outcome of PPV with or without ILM-peeling have been conducted and published in 2015 [40], 2016 [42] and 2017 [36]. They analysed data gathered over, respectively, 11, 11 and 13 years and from different hospitals. While Rayat et al. examined the outcome of different techniques of 32 eyes with ODP-M, only six received an ILM-peeling [40]. In the study from 2016 conducted by Steel et al. of 36 examined eyes with ODP-M, only nine got an additional ILM-peeling [42]. Avci et al. evaluated the data from 51 eyes treated by vitrectomy including 18 with additional ILM-peeling gathered from 13 different centres [36]. Recently, a new 2-year nationwide prospective population-based study by Steel et al. has been published [9]. While analysing the outcome of in total 25 patients with ODP-M managed by vitrectomy, 13 were treated with additional ILM-peeling and the surgical technique equally varied (9 with temporal juxtapapillary laser applied, 2 with SRF drainage, 24 with different gas tamponades, 1 with an ILM flap performed and 2 with inner retinal fenestration). Compared to the number of patients additionally treated with ILM-peeling observed in the others studies, our number of patients of ten is quite remarkable considering the monocentric design.

Only a prospective, carefully planned design might allow for a better standardization of the surgical techniques. A large prospective study with a longer follow-up time and a larger sample size, possibly multicentred, should be conducted to further investigate the effectiveness of gas tamponade and possible smaller advantages and disadvantages of ILM-peeling, including postoperative, full-thickness macular hole formation. However, the implementation of such a study is made more difficult by the rarity of the disease and the young age of many patients.

## Conclusion

Our results suggest that pars plana vitrectomy and gas tamponade are an effective treatment option for ODP-M, although the therapy is not successful in every case and ODP-M can reoccur after surgery. Additional ILM-peeling did not make a significant difference during initial surgery and may be a risk factor for the formation of full-thickness macular holes. However, all studies dealing with ODP-M surgical treatment have to cope with unavoidable systematic variations regarding surgical techniques, location and distribution of intraretinal fluid and long observation periods, which makes it hard to derive final conclusions.
