# Correction to: Surgical outcomes in patients with optic disc pit maculopathy: does peeling the ILM lead to better outcomes?

**DOI:** 10.1007/s10792-021-01942-7

**Published:** 2021-09-07

**Authors:** Helena Wagner, Amelie Pielen, Hansjürgen Agostini, Daniel Böhringer, Wolf Alexander Lagrèze, Julia Biermann

**Affiliations:** 1 Eye Center at Medical Center, University of Freiburg, Killianstrasse 5, Freiburg, Germany; 2 Faculty of Medicine, University of Freiburg, Freiburg, Germany; 3 Hannover Medical School, University Eye Hospital, Carl-Neuberg-Str. 1, Hannover, Germany; 4 Department of Ophthalmology, University of Muenster Medical Center, Domagkstrasse 15, 48149 Muenster, Germany

## Correction to: Int Ophthalmol (2020) 40:3363–3376 https://doi.org/10.1007/s10792-020-01524-z

The article “Surgical outcomes in patients with optic disc pit maculopathy: does peeling the ILM lead to better outcomes?”, written by Helena Wagner, Amelie Pielen, Hansjürgen Agostini, Daniel Böhringer, Wolf Alexander Lagrèze, Julia Biermann, was originally published Online First without Open Access. After publication in volume 40, issue 12, page 3363–3376, the author decided to opt for Open Choice and to make the article an Open Access publication. Therefore, the copyright of the article has been changed to © The Author(s) 2020, and the article is forthwith distributed under the terms of the Creative Commons Attribution 4.0 International License, which permits use, sharing, adaptation, distribution and reproduction in any medium or format, as long as you give appropriate credit to the original author(s) and the source, provide a link to the Creative Commons licence, and indicate if changes were made. The images or other third party material in this article are included in the article’s Creative Commons licence, unless indicated otherwise in a credit line to the material. If material is not included in the article’s Creative Commons licence, and your intended use is not permitted by statutory regulation or exceeds the permitted use, you will need to obtain permission directly from the copyright holder. To view a copy of this licence, visit http://creativecommons.org/licenses/by/4.0. Open access funding enabled and organized by Projekt DEAL.

The original article has been corrected.

